# Human parainfluenza virus fusion complex glycoproteins imaged in action on authentic viral surfaces

**DOI:** 10.1371/journal.ppat.1008883

**Published:** 2020-09-21

**Authors:** Tara C. Marcink, Tong Wang, Amedee des Georges, Matteo Porotto, Anne Moscona

**Affiliations:** 1 Department of Pediatrics, Columbia University Vagelos College of Physicians & Surgeons, New York, New York, United States of America; 2 Center for Host-Pathogen Interaction, Columbia University Vagelos College of Physicians & Surgeons, New York, New York, United States of America; 3 Structural Biology Initiative, CUNY Advanced Science Research Center, New York, New York, United States of America; 4 Department of Chemistry and Biochemistry, City College of New York, New York, New York, United States of America; 5 Department of Experimental Medicine, University of Campania "Luigi Vanvitelli", Naples, Italy; 6 Department of Microbiology & Immunology, Columbia University Vagelos College of Physicians & Surgeons, New York, New York, United States of America; 7 Department of Physiology & Columbia University Vagelos College of Physicians & Surgeons, New York, New York, United States of America; Institut Pasteur, FRANCE

## Abstract

Infection by human parainfluenza viruses (HPIVs) causes widespread lower respiratory diseases, including croup, bronchiolitis, and pneumonia, and there are no vaccines or effective treatments for these viruses. HPIV3 is a member of the *Respirovirus species* of the *Paramyxoviridae family*. These viruses are pleomorphic, enveloped viruses with genomes composed of single-stranded negative-sense RNA. During viral entry, the first step of infection, the viral fusion complex, comprised of the receptor-binding glycoprotein hemagglutinin-neuraminidase (HN) and the fusion glycoprotein (F), mediates fusion upon receptor binding. The HPIV3 transmembrane protein HN, like the receptor-binding proteins of other related viruses that enter host cells using membrane fusion, binds to a receptor molecule on the host cell plasma membrane, which triggers the F glycoprotein to undergo major conformational rearrangements, promoting viral entry. Subsequent fusion of the viral and host membranes allows delivery of the viral genetic material into the host cell. The intermediate states in viral entry are transient and thermodynamically unstable, making it impossible to understand these transitions using standard methods, yet understanding these transition states is important for expanding our knowledge of the viral entry process. In this study, we use cryo-electron tomography (cryo-ET) to dissect the stepwise process by which the receptor-binding protein triggers F-mediated fusion, when forming a complex with receptor-bearing membranes. Using an on-grid antibody capture method that facilitates examination of fresh, biologically active strains of virus directly from supernatant fluids and a series of biological tools that permit the capture of intermediate states in the fusion process, we visualize the series of events that occur when a pristine, authentic viral particle interacts with target receptors and proceeds from the viral entry steps of receptor engagement to membrane fusion.

## Author summary

Human parainfluenza viruses (HPIVs) cause the majority of childhood cases of croup, bronchiolitis, and pneumonia. HPIV3, like most other paramyxoviruses, uses two specialized proteins to mediate cell entry: the fusion protein (F) and the receptor-binding protein, hemagglutinin-neuraminidase (HN). F is only activated to mediate membrane fusion during entry when it is triggered by a signal from the separate receptor-binding molecule, HN–a mechanism first elucidated for HPIV3 and later extended to this entire group of human pathogenic viruses. These two distinct membrane glycoproteins work in synchrony as a molecular machine, exquisitely tuned to enter the right cells at the right time. Much has been learned about the functional sites on the receptor-binding and F molecules for this group of viruses, and mechanisms have been proposed to explain molecular, biochemical, and crystal structure data. But the key intermediate steps, from receptor binding to membrane fusion, are fleeting and unstable. Furthermore, it is unclear how HN ultimately promotes F-mediated fusion. Thus, it is impossible to understand intermediate fusion states, using standard methods and static observations. Here, we use an on-grid antibody capture method that facilitates examination of fresh, biologically active strains of virus directly from supernatant fluids by cryo-electron tomography and new biological tools that permit capture of the intermediate states in the fusion process. It allows us to visualize the series of events that occur as an authentic HPIV3 viral particle interacts with target receptors and proceeds through intermediate stages from receptor engagement to membrane fusion.

## Introduction

Human parainfluenza virus (HPIV) entry into human airway epithelial cells, as the initial step of infection, is mediated by fusion of viral and host cell membranes at the cell surface. HPIV3 is a member of the *Respirovirus* species of the *Paramyxoviridae* family and is a pleomorphic, enveloped virus with a genome composed of single-stranded negative-sense RNA. The 3-dimensional ultrastructure of HPIV3 virions in the absence of receptor engagement has been previously characterized [1]. Virus-cell fusion for parainfluenza, as well as for most other enveloped RNA viruses of the Paramyxovirus family [2,3], results from the coordinated action of the two envelope glycoproteins that comprise the viral entry complex—the receptor binding protein (hemagglutinin neuraminidase (HN) for HPIV3) and a separate membrane fusion protein (F). This well-timed cooperation between two separate surface glycoproteins is different than the fusion machinery of influenza virus, wherein just the hemagglutinin protein contains both the receptor-binding and the fusion domains. Instead, the HPIV3 envelope glycoproteins, HN and F, form a fusion complex and work together to mediate viral attachment and entry into host cells. While the exact receptor is unknown, the HPIV3 transmembrane protein HN binds preferentially to a α2,3-linked sialic acid-containing receptor [4,5] on the host cell plasma membrane, and the F protein, once activated by the receptor-binding protein after receptor engagement [6–8], mediates the fusion of viral and host membranes, in order to deliver the viral genetic material into the host cell.

Paramyxovirus F proteins are synthesized as precursors (F_0_) that are cleaved within the cell to yield the pre-fusion F trimer with F_1_ and F_2_ remaining covalently linked via a disulfide bond [9,10]. This trimeric F structure is present on the surface of an infectious viral particle in a metastable pre-fusion conformation with the hydrophobic “fusion peptide” buried in the interior of the molecule. However, once the F protein undergoes a major structural transition, the hydrophobic fusion peptide emerges from its protected site, inserting into the host membrane when the appropriate biological trigger is sensed, ideally upon contact of the receptor binding protein with the host cell and then F converts to a highly stable post-fusion form [11–16]. Active participation of receptor-engaged HN is required for the F-mediated fusion process of HPIV3 [8,17,18]. In addition, the engagement of other paramyxovirus receptor-binding proteins (HN for HPIV-1,-2,-3,-4; hemagglutinin (H) for measles; G glycoprotein for Nipah, or Hendra virus) with their respective receptors are necessary to induce conformational changes in their respective F proteins, resulting in fusion of the viral and cellular membranes [2,3,16,19–23]. After activation, when F proceeds to refold into its energetically stable post-fusion structure, as the N-terminal and C-terminal complementary heptad repeats meet to form a stable six-helix bundle, the refolding drives fusion of the viral and cell membranes and release of the viral genetic material into the target cell [12,13,15,16,24–30].

HPIV3 fusion machinery has primarily been studied in cell biological assays [19,31–35] or in biochemical settings, removed from natural systems [36–38]. In previous studies using viral glycoproteins expressed in cultured monolayer cells, we showed that the HPIV3 HN and F proteins interact before and during fusion activation [34] and observed the sequence of events leading up to HN-F-mediated membrane fusion in real-time in live cells, using bimolecular fluorescent complementation, a technique that allows HN-F interactions to be studied under biological conditions [11,33,34,39–41]. We showed that HN’s engagement with receptor molecules drives the formation of HN-F clusters at the site of fusion and that a second sialic acid receptor-binding site, positioned in the dimer interface of HPIV3 HN (“site II”), directly modulates interaction with F and in turn F-activation in living cells [8,11,32,39,42]. After initial activation of F, HN and F remain associated, and HN acts on F, even beyond the step of fusion peptide insertion into the host membrane. As fusion progresses further, either HN or F dissociate from the complex, or the clusters of HN-F complexes disperse [11].

While well-characterized for influenza [43–46] and HIV [47–52], the structural organization of glycoproteins and their architecture on HPIV3 viral surfaces is largely unknown [1,11]. The 3-dimensional structure of the F fusion trimer from multiple paramyxoviruses, including HPIV3, has been described in both the pre-fusion and post-fusion forms [12,53–56]. The F monomers oligomerize into a squat trimer with a central cavity, giving it a rounded appearance from the side and a triangular shape when viewed from above [13,57]. The ectodomain sits on top of a short tether, formed by portions of the C-terminal heptad repeat (HRC) segments, which are anchored via the transmembrane domain to the viral membrane. Recently, the structure of HPIV3 pre-fusion F was solved by cryo-electron microscopy (EM) [53]. This modified structure included multiple non-natural disulfide bonds, along with other mutations to stabilize the soluble form. Currently, there are no proposed structures of either authentic full-length HN or F inserted into lipid bilayer membranes, i.e. in their natural state.

The activities of HN–receptor binding, receptor cleaving, fusion activation, and possibly F protein stabilization–are regulated at specific points during the viral life cycle. The stalk of HN confers specificity for the homologous F in the fusion activation process [8,58–63]. The primary binding/neuraminidase active site residues are located on the globular head of HN for HPIV3 and for the receptor-binding protein of other paramyxoviruses for which crystal structure information is available [64–67]. For Newcastle disease virus (NDV), HPIV1, and HPIV3, a secondary sialic acid binding site on HN plays a distinctive role in receptor-binding and/or promoting fusion [8,32,39,42,65,68]. Receptor-binding proteins from various paramyxoviruses have been characterized using crystallography in several conformations and oligomeric forms [8,22,42,58,66,67,69–75]. These methods have led to the suggestion that these proteins can adopt at least three arrangements, including a “heads-down” tetramer in which two dimers of HN are organized around a 4-helix stalk [58], a “heads-up” tetramer form [67], and a two “heads-up” two “heads-down” form that has been proposed to represent an intermediate conformation [69]. However, it has not previously been possible to probe these molecules directly, using unperturbed, authentic virions or to use biologically relevant viruses to test the models of activation that have emerged from experimental data [2,3,6,11,16,19–23,34,42].

Cryo-EM has been previously employed to examine the architecture of paramyxoviruses, including measles virus [76–78], canine distemper virus [79], Sendai virus [80–81], respiratory syncytial virus [82–84], and simian virus 5 /parainfluenza virus 5 (SV5/PIV5) [85,86]. We previously used cryo-electron tomography (cryo-ET) to image the 3-dimensional architecture of HN-F complexes on the surfaces of virions, prior to receptor engagement [1]. For those experiments, we used viral particles that had been processed similarly to many viral particles studied by EM: purified by ultracentrifugation. We showed that, prior to receptor engagement, HN and F are associated with each other on the surface of virions and that F in complex with HN is present in its pre-fusion conformation [1]. However, until now, it has not been possible to image the series of events that occur *after* HN binds to the receptor, and F is activated by HN to fuse, as a hydrophobic region of F (the fusion peptide) emerges from its protected site, in order to insert into the host membrane. Then, the F protein rearranges and becomes elongated, and the protein folds back onto itself to form a “hairpin”, and fusion proceeds, resulting in merging of the viral and cellular membranes. These intermediate states in viral entry are transient and thermodynamically unstable, making it impossible to understand these transitions using standard methods. Images of the simian virus 5 / parainfluenza virus 5 (SV5/PIV5) fusion protein intermediate state, triggered by heat and captured with a fusion-inhibitory peptide, were examined using negative stain EM [85]; more recently, multiple intermediate states of influenza hemagglutinin (HA), including the first structures of an extended trimer state, were solved using cryo-EM [43,87]. These rarely visualized transition states are significant for fundamental understanding of the viral entry process.

Advances in grid affinity purification techniques have improved the ability to capture and image challenging biological specimens [88,89]. Nickel-NTA lipids adhered to cryo-EM grids have been used to capture pleomorphic viruses, including measles and influenza viral particles [88]. Coating grids with antibodies as an approach to capture viral particles for purification and visualization was first described in 1973 for the detection of plant viruses [90]. This on-grid antibody technique has since been adapted for use in the detection of rotaviruses [91] and noroviruses [92,93] in patient samples. Recently, this technique was adapted for use in cryo-EM by Yu et. al (2016) for imaging the glycoprotein complexes of several viruses, including the Sindbis and Tulane viruses [94,95]. Here, we used an on-grid antibody capture method that permits examination of fresh, biologically active strains of virus directly from supernatant fluids, combined with a series of new biological tools to visualize the events that occur as viral particles interact with target receptors, proceeding stepwise from receptor engagement to membrane fusion.

## Results

### Capture of authentic viral particles avoids purification artifacts

Previous studies in our laboratory used viral preparations that had been ultracentrifuged to concentrate the viruses, using standard methods [1], requiring several manipulation steps. These steps could undermine capture of native states. We adapted a method that permits the capture of fresh virus directly from cellular supernatant fluid with minimal manipulation [94,96]. In our method, the grids are coated with an anti-HN antibody at room temperature, and supernatant fluid containing virus is added directly at any desired temperature. The experimental conditions of interest (*e*.*g*., compound incubation or exposure to cellular membranes) can be carried out directly on these grids. This method can be performed in tandem with other biological assays, allowing correlation between biological and structural data. In this way, the viral particles are not subjected to manipulation that could introduce potential artifacts. The on-grid antibody capture of viral particles directly from cell culture supernatant fluid eliminates the need for purification and yields clean grids with authentic viral particles that are intact and infectious.

### HPIV3 particle morphology

**Fig 1:**
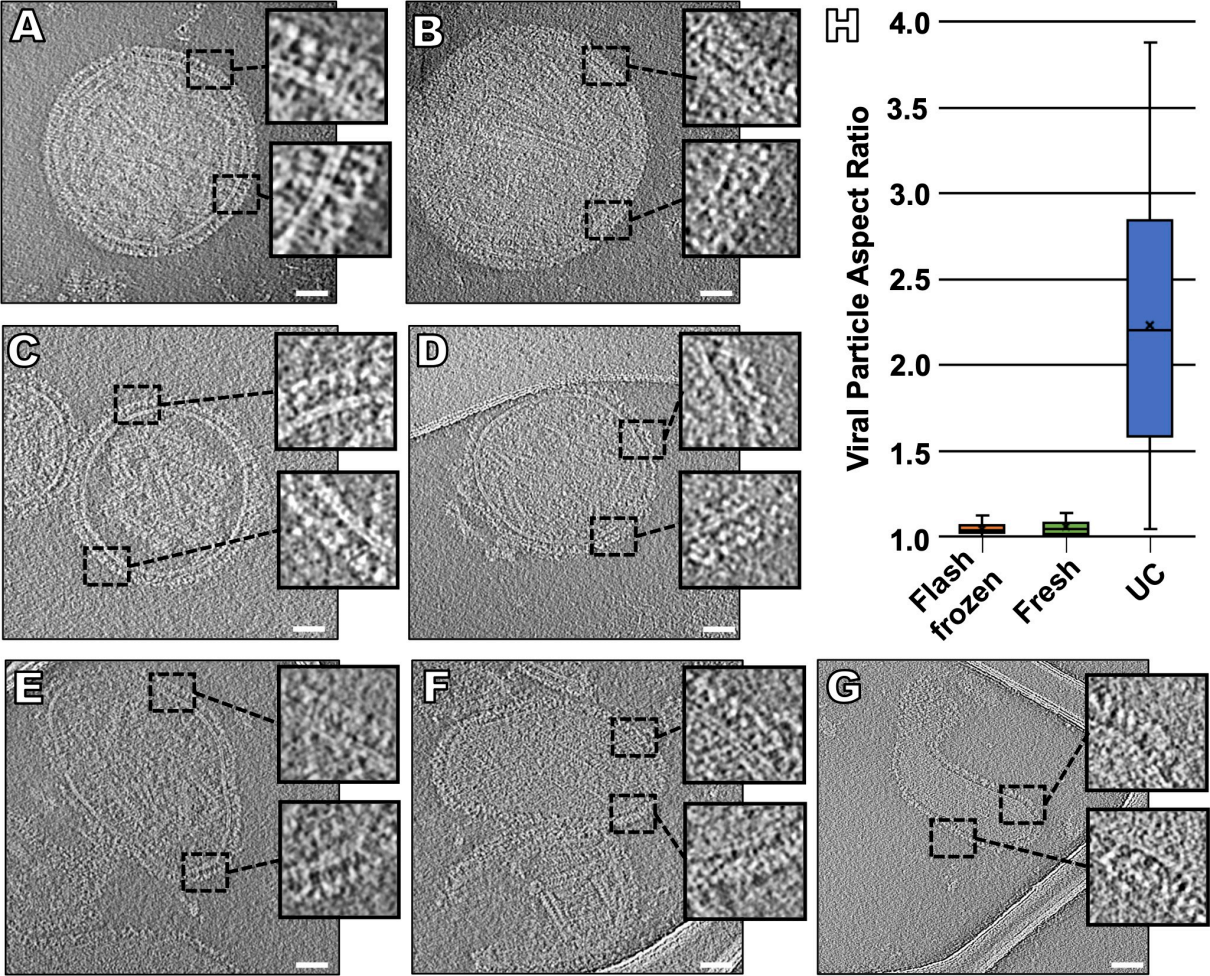
Changes in viral morphology resulting from different purification methods. Contrast inverted cryo-ET central slices of clarified supernant fluid collected directly from infected Vero cells and either imaged after being flash frozen (**A, B**), fresh (**C, D**), or ultracentrifuged (**E-G**). Inserts represent enlarged regions of the viral glycoprotein layer. (**H**) Viral particle aspect ratio of all three purification methods (n = 50). Scale bars: (**A-G**) 50 nm.

In previous ultrastructural studies, paramyxoviruses were observed to be pleomorphic, as examined using both negative-stain and cryo-EM and cryo-ET [76,80,82,86,97,98]. In our previous cryo-ET study, where we used standard ultracentrifugation methods for viral preparation [1], we observed a combination of elongated and spherical particles. The vast majority of particles (~90%) that we observed appeared to lack a membrane-associated matrix layer, and those that did exhibit thicker envelopes with an internal matrix layer appeared to be generally smaller particles, sometimes with discernable post-fusion F proteins on their surfaces [6,99]. In contrast, using the on-grid antibody capture method, we found that, while viral particles still showed similarities in their diameter, lipid bilayer and lack of matrix organization, >95% of the viral particles were spherical, prior to receptor engagement or fusion (**Fig 1**). We did not observe the panoply of diverse viral shapes observed in previous studies, which utilized processed virions [1,77,100]. Using this method, we obtained clean images of individual HPIV3 particles free of cellular debris, and >95% of the viral particles imaged contained a dense glycoprotein coat on all the viral surfaces (**Fig 1A and 1B**). Of note, the viral particles that we had frozen and thawed once had the same surface glycoprotein organization as those that were captured and vitrified directly from fresh supernatant fluid (**Fig 1C and 1D**), and all samples had irregular matrix protein layers, as seen with NDV particles after budding [98]. When we applied this on-grid antibody capture method to ultracentrifuged viral particles (**Fig 1E–1G**), those particles lacked glycoprotein arrays and contained shape distortions, indicated by a high aspect ratio, confirming that the deviations from authentic viral particle morphology are related to the prior purification techniques (**Fig 1H**) [101].

**Fig 2:**
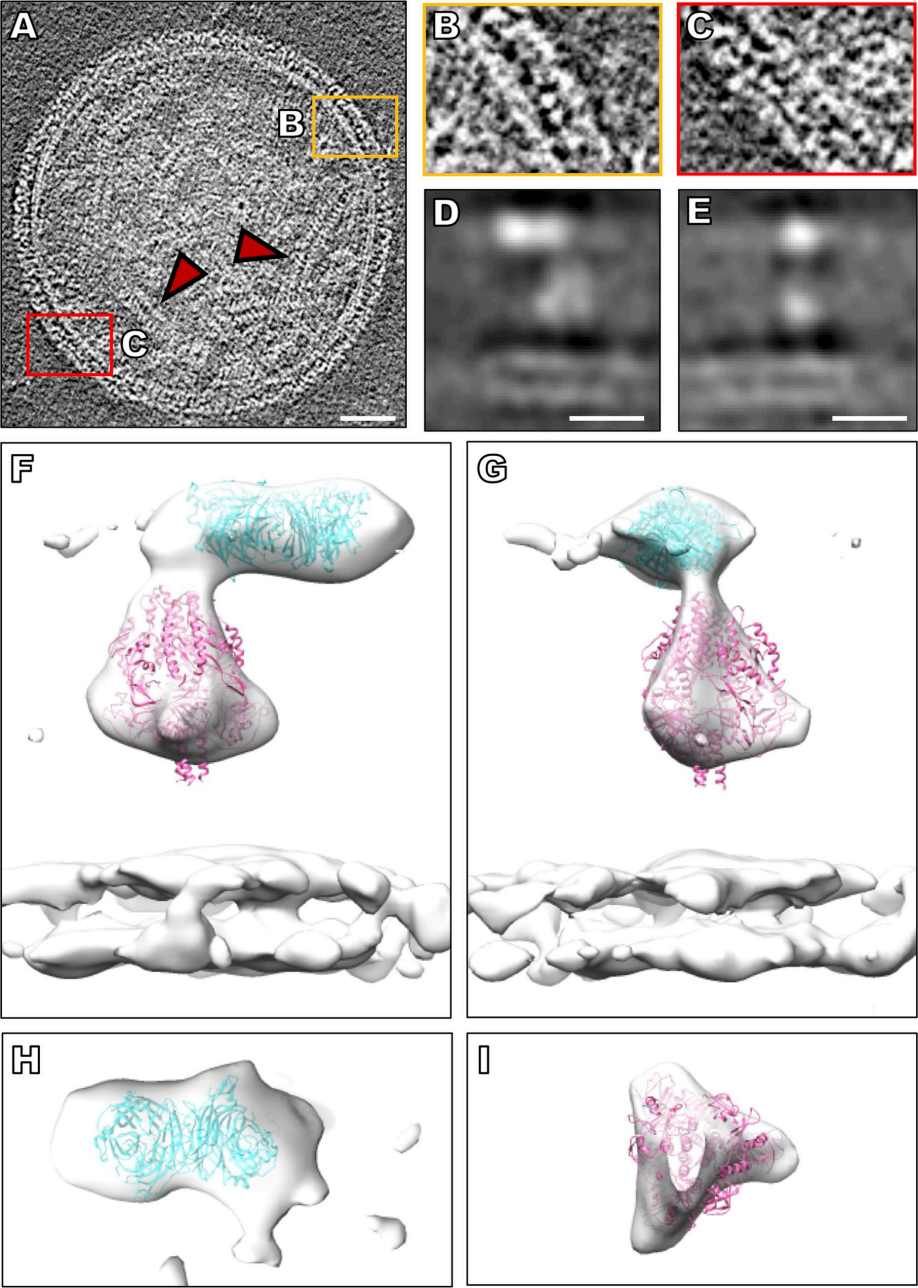
Viral tomographic reconstructions. (**A**) Contrast inverted cryo-ET central slice of HPIV3 before receptor engagement. Red arrows indicate ribonucleoprotein helical tubes. (**B, C**) Enlarged regions of the surface glycoproteins with HN and F in tight arrangement. (**D, E**) Central (**D**) X slice and (**E**) Y slice through the subtomogram average of HPIV3 surface glycoproteins. (**F, G**) Sub-volume average of surface glycoproteins with crystal structure of the HN dimer (PDB ID: 4MZA) and the cryo-EM structure of pre-fusion F (PDB ID: 6MJZ) in green and pink (respectively), fitted into the sub-volume average. (**H**) Top-down view of HN and (**I**) top-down view of F. Scale bars: (**A**) 50 nm and (**D, E**) 10 nm.

Tomograms obtained using the on-grid antibody capture method show that the viral surfaces are wrapped in two primary layers of density (**Fig 2A**, **S1 Movie**). These densities correspond to pre-fusion F molecules that form a lower layer **(Fig 2A)** adjacent to and below HN molecules. Half-maximal distance from the membrane for HN is approximately 17 nm, and this distance is approximately 12 nm for F (**Fig 2B and 2C**), in line with previously measured distances [1]. However, in the current images of on-grid antibody-captured virions, the double layered areas, composed of HN molecules and pre-fusion F molecules, cover the observable viral surface, and we did not observe patches of arrays with HN or F alone, as previously noted with standard purification methods, including centrifugation [1]. To better resolve the complex of HN and F, we used sub-volume averaging (**Fig 2D–2I** and **S1A Fig**) and obtained a complex of HN and F at a resolution of 17 Å (**S1B–S1D Fig**). 2D slices through the middle of the sub-volumes show defined features for HN, F, and the membrane (**Fig 2D and 2E**). Fitting prefusion F (PDB ID:6MJZ) [53] and HN dimer (PDB ID:4MZA) [42] structures into the 3D sub-volume averages further supports the idea that these densities in the original tomograms were HN and F complexes (**Fig 2F and 2G**). The top-down view of the HN region shows a density consistent with an HN dimer (**Fig 2H**), while the top-down view of the F region represents a trimeric-shaped density (**Fig 2I**). To further assess the oligomeric state of HN, we fitted two PIV5 HN crystal structures, one with both HN dimers in a heads-up position (PDB ID:1Z50) [67] and the other with one HN dimer in a heads-down position (PDB ID:4JF7) [69] in the sub-volume averages. Neither structures matched the observed density (**S2 Fig**). For the paramyxovirus NDV, examination of HN ectodomains that included the stalk domains revealed primarily monomers and dimers in solution [58]. Our data strongly suggest that HN exists primarily as a dimer at the surface of HPIV3, prior to receptor engagement, and that it is in a “heads-up” conformation.

### HPIV3 particles imaged in contact with target cell receptors: Viral entry step 1

**Fig 3:**
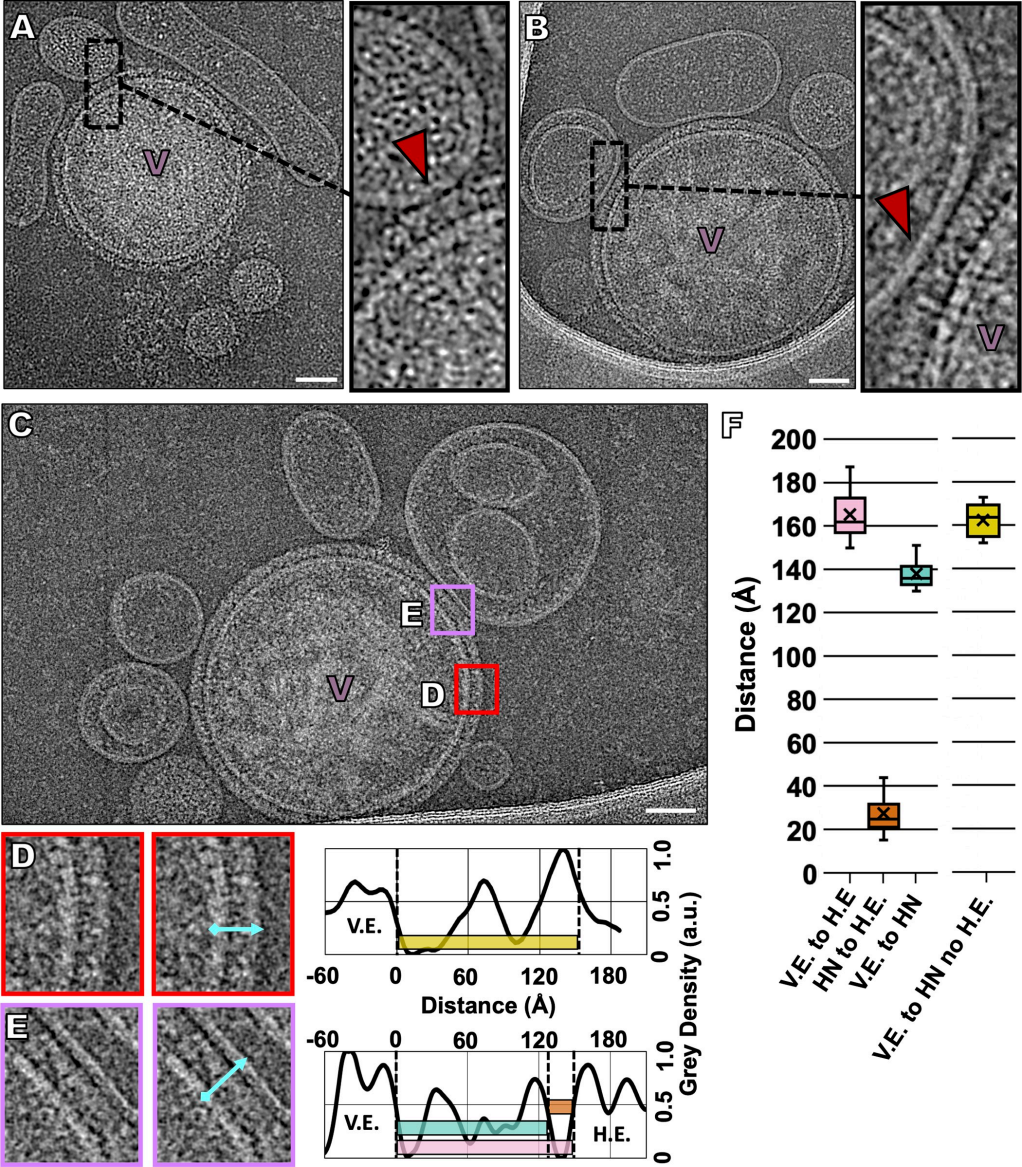
Imaging the interaction of the HPIV3 virions with a natural host receptor. To capture viral-host interactions and prevent the activation of F, samples were incubated at 4°C prior to vitrification. (**A, B, C**) Contrast-inverted (**A**) cryo-ET and (**B, C**) cryo-EM images of HPIV3 interactions with target erythrocyte fragment membranes. Insets: enlarged areas show thin lines of density connecting HN to the target erythrocyte fragment membranes. (**D, E**) Enlarged regions (**D**) distal and (**E**) proximal to the viral-host interaction site with accompanying density line plots of each region. Relative density measurements in arbitrary units (a.u.) of the space between the viral envelope (V.E.) and HN or the target human erythrocyte fragment membrane (H.E.) with distances measured from positions of half-maximum density (distance at half-maximum density of outer leaflet to peak intensity distal to membrane), showing the positions of each viral envelope glycoprotein. (**F**) Box plot of distances measured from regions equivalent to (**D**) and (**E**) with each distance represented by boxes of the same color as those overlaid in the graphs of panels (**D**) and (**E**). Dashed vertical lines indicate half maximum distance of the viral envelope (V.E.), HN, and host envelope (H.E.). Numbers indicate average heights of 38 measurements with +/- standard deviations. Scale bars: (**A, B, C**) 50 nm.

In order to image the viral surface interacting with natural receptor molecules without progression to fusion, receptor-bearing erythrocyte fragments were exposed to virus captured on grids at 4°C (**Fig 3, S3 Fig, S2 Movie**). Erythrocyte-derived membranes contain the sialic acid receptors used by HPIV3 [102], as well as a reasonably authentic lipid composition, similar to typical plasma membranes [102]. We previously showed that presenting receptor in this manner allows efficient F protein activation and fusion [33,34]. While HPIV3 does not infect erythrocytes in humans, erythrocyte fragment membranes can provide an excellent surrogate lipid bilayer cell membrane. This approach allows us to avoid using an artificial composition of lipids and glycolipids (used in synthetic liposomes) in our target membrane. After incubating the grids with antibody (see the methods section), the grids were placed on top of a mixture of supernatant fluid containing both viral particles and target erythrocyte fragment membranes at 4°C. After 30 minutes, the grids were washed with Dulbecco's modified phosphate-buffered saline (DPBS) to ensure that predominantly target erythrocyte fragment membranes that had attached to the viral particles will remain on the grids. At 4°C, the viruses bind to target membranes, but F cannot be activated [34,41]. This stage represents the first step in entry, binding of HN to cellular receptors, and F should maintain a pre-fusion state [11,34]. Our results showed viral particles closely bound to target erythrocyte fragment membranes (**Fig 3** and **S3A–S3D Fig**). As the sample was incubated at 4°C to inhibit F insertion into the target erythrocyte fragment membrane, these viruses should only be attached to the target membrane via the engagement of HN with its target receptor. To test this hypothesis, we repeated our experiment at 4°C with the addition of 2 mM zanamivir, a small molecule that prevents HN-receptor engagement [103], after the initial incubation of viruses with target erythrocyte fragment membranes **(S3E–S3H Fig)**. As expected, almost no target erythrocyte fragment membranes could be found interacting with viral particles; the images show that, even where target erythrocyte fragment membranes are present, they do not contact viral particles. This shows that, even after the erythrocyte fragment membranes attach to viral glycoproteins, the addition of zanamivir detaches them from the virus, consistent with previous results [34] and with the idea that at 4°C these viral particles attach to host membranes via HN-receptor engagement alone and not via F insertion into the host membrane. Further supporting this notion, we observe the characteristic Y-shaped density of HN at several virus-erythrocyte fragment contact points, with thin lines of density extending from the HN head to the target erythrocyte fragment membrane (**Fig 3** and **S3 Fig**), which we suggest are HNs engaged with their receptor.

To assess the geometry and conformation of the glycoproteins at areas of virus-erythrocyte fragment contact and away from these areas, we measured the size of structures on the viral membrane (**Fig 3D, 3E and 3F**). Distal to the viral-erythrocyte fragment region of contact, HN measures approximately 163 Å from the viral surface (**Fig 3D and 3F)**, while F measures 90–120 Å, consistent with previous values for HN and pre-fusion F [1]. In regions of contact between the viral and target erythrocyte fragment membranes, the average distance between the viral membrane and the HN heads is 138 Å (**Fig 3E and 3F,** teal). Intriguingly, this is 25 Å less than the distance from the viral membrane to the HN head, in absence of the host receptor. This difference suggests that HN undergoes some adjustment after receptor engagement. Further supporting this notion, the distance between the viral membrane and the target erythrocyte fragment membrane in areas of contact is only 165 Å (**Fig 3E and 3F**, pink). HN in the conformation measured in the absence of target erythrocyte fragment membrane (**Fig 3E and 3F**, yellow) would not fit in such space without rearrangement. Furthermore, at points of contact, we observe that the average distance between the HN heads and the target erythrocyte fragment membrane is 27 Å (**Fig 3E and 3F,** orange). This significant gap is not consistent with a conformation of HN constrained by steric clash with the target erythrocyte fragment membrane at the stage visualized here.

### Capture of the transient intermediate state of F, extended and inserted into the target membrane: viral entry step 2

**Fig 4:**
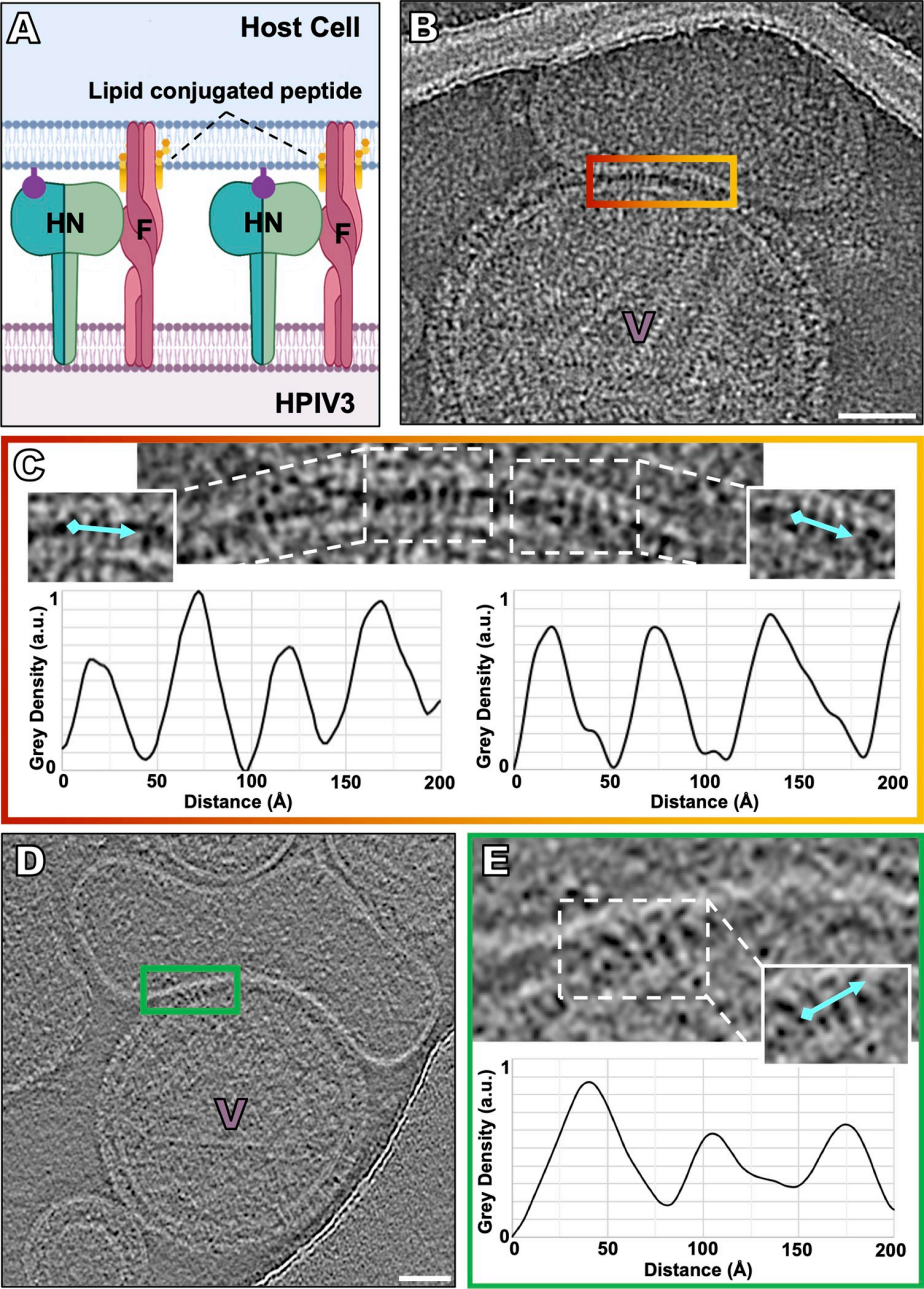
Capture of the transient intermediate state of F with lipid-conjugated fusion inhibitory peptides. To capture the transient intermediate fusion state just after HN activates F, receptor-bearing target erythrocyte fragment membranes were exposed to virus on grids at 37°C in the presence of lipid conjugated fusion inhibitory peptides *(i*.*e*., VIKI-PEG_4_-chol), prior to vitrification. (**A**) Schematic of lipid-conjugated peptides inserting into the target cell membrane via their lipid tails and “locking” the extended F in its transient intermediate state, preventing refolding to the post-fusion conformation. (**B, D**) Contrast-inverted images where viral particles can be observed attached to target erythrocyte fragment membranes using (**B**) cryo-EM and (**D**) cryo-ET. (**C, E**) Enlarged region of interactions between the viral and target erythrocyte fragment membranes where elongated densities linking both membranes are visible. Insets include cyan lines where distance plot measurements were taken. **(C,D,E, bottom)** Density line plots revealing a repeating 20–35 Å-wide density. Scale bars: (**B, E**) 50 nm.

To observe the next step in viral entry, the temperature was warmed to allow activation of F to occur, while progress towards fusion was blocked using VIKI-PEG_4_-chol, a peptide that corresponds to the C-terminal heptad repeat (HRC) region of F protein [85,104–109] and binds to the extended intermediate states of F [41,110] (**Fig 4, S4 and S5 Figs, S3 Movie**). To capture the transient intermediate fusion state just after HN activates F, receptor-bearing target erythrocyte fragment membranes were exposed to virus on grids at 37°C, in the presence of the fusion inhibitory peptide VIKI-PEG_4_-chol [33]. The peptide inserts into the target cell membrane via lipid tails and binds to the N-terminal HR regions of F, “locking” the extended F in its transient intermediate state by blocking interaction of the N-terminal HR with its C-terminal complement, thereby preventing refolding to the post-fusion conformation [33] (schematically diagrammed in **Fig 4A**). At this temperature, HN can activate F [33–34], and, if HN-receptor engagement is permitted, the HN-receptor interaction will trigger F, and the peptide will interact with the N-terminal HR region of F [33]. At 37°C, in the presence of the lipid-conjugated fusion inhibitory peptide (VIKI-PEG_4_-chol), viral particles can be observed attached to target erythrocyte fragment membranes (**Fig 4B–4D and S4 Fig**) with densities crossing the space between the viral surface and the target erythrocyte membrane (**Fig 4C–4E**). The densities that extend from the viral membrane to the target erythrocyte fragment membrane do not correspond to either HN or to the pre-fusion F (**as seen in Fig 2A–2C)** and are distinct from the observed configuration of F during receptor engagement at 4 ^o^C (**as seen in Fig 3A–3E)**. Density profiles of these regions reveal palisade-like densities with diameters estimated to be between 19 and 30 Å (**Fig 4C–4E and S4 Fig**). In the presence of the fusion inhibitory peptide (VIKI-PEG_4_-chol), which halts the progression of fusion past the insertion of F into the target membrane, no fused particles were observed. When zanamivir, the small molecule that disrupts HN-receptor interaction, is added, the thin lines of density between the viral membrane and target erythrocyte fragment membrane do *not* detach, confirming that the interaction between the membranes is not merely due to HN-receptor binding but via F insertion into the target erythrocyte fragment membrane [8,34] **(S5D–S5F Fig)**. In the presence of the fusion inhibitory peptide VIKI-PEG_4_-chol, we also observed viral-host lipid bilayer contact **(S5A–S5C Fig)** and weak density near viral-erythrocyte fragment membrane contact areas, suggesting potential lipid mixing **(S5B and S5C Fig)**.

### Progression to fusion with the target membrane: Viral entry step 3

**Fig 5:**
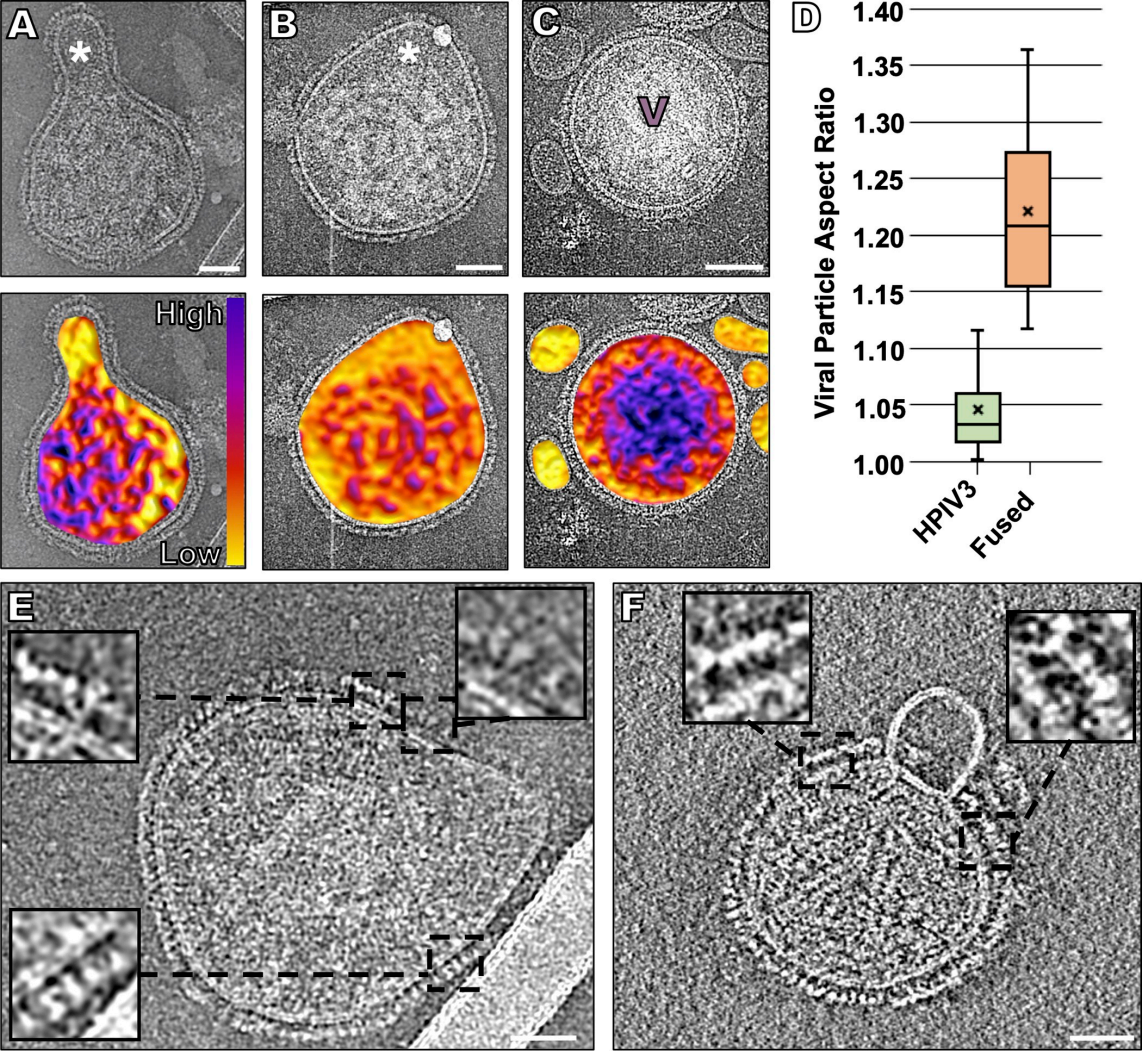
Fusion of HPIV3 with a target erythrocyte fragment membrane. (**A, B**) Contrast-inverted viruses that underwent fusion with target erythrocyte fragment membranes (top) with density color representation overlaid below. The purple color represents the dense viral ribonucleoprotein, and the yellow color represents the erythrocyte content. (**C**) Negative control where grids were kept at 4°C, prior to vitrification to prevent fusion of target erythrocyte membranes with the viruses. (**D**) Particle aspect ratio of viruses in the presence of zanamivir (also incubated at 4°C, as in **C**), compared to fused particles (n = 23). (**E**) Fusion with a small target erythrocyte fragment membrane reveals a lack of prefusion F density near the sites of fusion (inserts). (**F**) A possible instance of hemifusion where the target erythrocyte fragment membrane surface shows no evidence of surface glycoproteins, and the viral surface shows a lack of prefusion F density near the sites of fusion (inserts). Scale bars: (**A-C**) and (**E, F**) 50 nm.

In order to permit fusion to proceed, target erythrocyte fragment membrane were simply (*i*.*e*., without any inhibitors) exposed to virus on grids at 37°C. As shown in **Fig 5A and 5B**, these viruses underwent fusion with the target erythrocyte fragment membranes. The ribonucleoprotein content of the viral particle is much denser than the content of the target erythrocyte fragment membrane, and the portion of the structure that derives from each entity can be discerned by density differences that correspond to regions of the virus that have lost their sphericity (**Fig 5D**). To highlight those density differences, we represented content density using a color scale (**Fig 5A and 5B lower panels**). In contrast, when grids were kept at 4°C to prevent erythrocyte-virus fusion, viral particles retain their sphericity, and no density fluctuations are observed within them (**Fig 5C and 5D**). A physiological host target cell would clearly be far larger in diameter than these erythrocyte fragment membranes but would not be amenable to cryo-EM or cryo-ET analysis without the combined use of correlative light and electron microscopy and FIB-milling to identify and image virus-host interaction events. In **Fig 5E**, a sparse glycoprotein layer can be seen in areas where fusion has occurred. Insets of **Fig 5E** and **5F** indicate a lack of density in the lower glycoprotein layer, along with a density that appears to be post-fusion F (**Fig 5E and 5F insets**). Images of the hemifusion and early fusion pore states were rarely seen; one particle that we identified in a possible hemifusion state appears to have a 500 Å wide merged membrane (**Fig 5F**).

## Discussion

**Fig 6:**
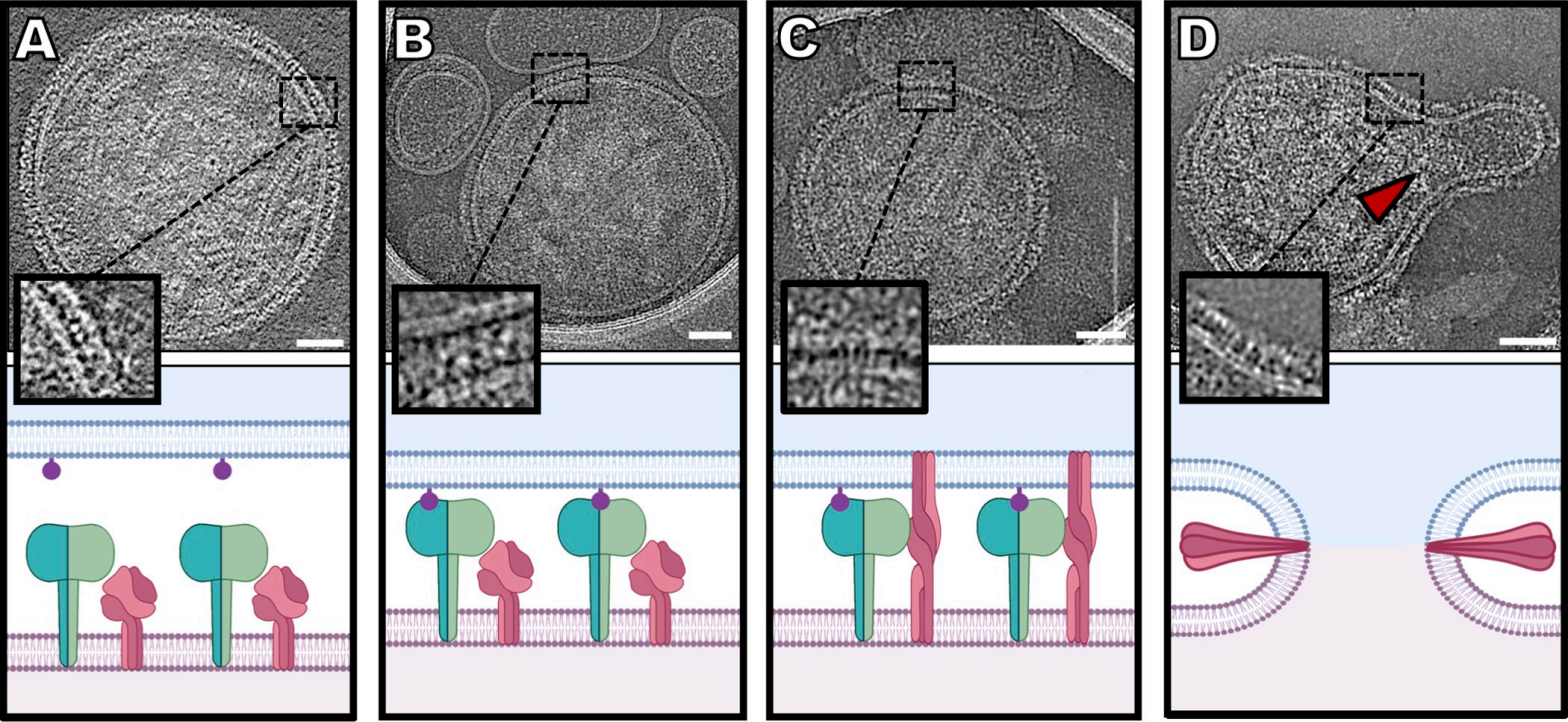
Sequence of events in HPIV3 entry, corresponding to cryo-electron microscopy imaging. (**A**) HN (green) and F (dark pink) can be found densely packed on the viral surface (light pink) (Image from **Fig 1A**). (**B**) Sialic acid (purple) binding to HN occurs in the presence of a host target membrane (blue) (Image from **Fig 3B**). (**C**) Upon triggering of F by HN, F undergoes a large conformational change from a pre-fusion globular structure to an extended structure that crosses both membranes (Image from **Fig 4B**). (**D**) After this intermediate state, F folds back onto itself, pulling both membranes towards each other, creating a pore in a process that ultimately results in a merged membrane. Scale bars: (**A-D**) 50 nm.

The cryo-ET and cryo-EM studies reported here provide images of glycoprotein organization on the surface of HPIV3 during the steps of viral entry and fusion, starting with receptor engagement and ending with fusion of the viral envelope with a target membrane (summarized in **Fig 6**). This process shows the ability to capture undisrupted virus that is intact and biologically active. Taken together with experimental data, these results form a basis for elucidation of the authentic entry mechanism of paramyxoviruses.

A set of biological tools that were generated and validated in biological, biochemical, and biophysical assays [6,11,33] have now permitted us to capture each of the steps of viral entry from viral receptor binding through fusion with the host membrane. These tools include small binding inhibitors (zanamivir) and lipid-conjugated fusion-inhibitory peptides (VIKI-PEG_4_-chol) that engage the fusion protein once extended but prevent it from refolding into its post-fusion state. The use of antiviral antibodies for isolating virus on EM grids without centrifugation and the presentation of a host target membrane surrogate on the grid made it possible to examine authentic viral particles interacting with target membranes. These strategies can be applied to studying a wide variety of viruses and other agents that interact with host target membranes, as well as biological assemblies and macromolecular complexes.

In contrast to previous images of parainfluenza viruses, where a variety of particle shapes, ranging from spherical to filamentous were noted [77,82,100], the authentic HPIV3 virions here are predominantly spherical. This leads us to speculate that perhaps this distinction that results from preparation method applies to other enveloped viruses as well. Filamentous and spherical viral particles of several related viruses, including measles, respiratory syncytial virus, and Newcastle disease virus, have been captured during the assembly process of authentic viruses using whole-cell tomography, but, of note, these images were captured during and immediately after viral egress [77,80,97,98]. While previous studies, including ours, showed both filamentous particles and irregular patches of glycoproteins on viral surfaces, including some patches of HN alone [1], the particles we observed here, in the absence of ultracentrifugation, were, in contrast, quite spherical and uniformly coated with a double layer of glycoproteins, comprised of HN and pre-fusion F. Ultracentrifugation can not only alter viral particle integrity, as has been shown for influenza, but also promote the artificial selection of viruses of a specific density or morphology [101].

Despite differences in viral shape and surface glycoprotein organization, we could not see ordered matrix protein under the viral membrane, regardless of preparation method. The disassociation of the matrix protein from the viral membrane is necessary for infection, and spherical particles with a dissociated matrix layer have been hypothesized to represent a primed infectious viral particle after budding [98]. We contend that the surface glycoprotein organization and stability observed in the past may be due to artifacts generated by purification approaches. Of note, our previous study used the CV-1 cell line for viral propagation, instead of the Vero cell line used in this study, which could theoretically affect the morphology of the viral particles, despite the fact that both cell lines are derived from African green monkey kidneys. However, we observed that the ultracentrifuged particles propagated in Vero cells in this study have identical characteristics as ultracentrifuged particles obtained from CV-1 cells, including differences in surface glycoprotein organization and viral shape (**Fig 1**), suggesting that the process of ultracentrifugation, not the specific cell line used, promotes alterations in virion morphology. Future studies that employ the on-grid antibody capture methods will examine whether surface glycoprotein features differ between viruses that are collected from cell lines and isolates from *ex vivo* tissues and/or *in vivo*.

Previous structural studies have identified HN as existing in several potential oligomeric configurations, including a heads-down conformation, prior to sialic acid binding [3] implying that an HN heads-up conformation would occur upon receptor engagement. Early cross-linking studies suggested that HPIV3 HN could exist as a tetramer [111], although crystal structures of HPIV3 HN have only shown the dimeric form [66]. Here, prior to receptor engagement, we found HN only as a dimer and only with the globular heads extended above the stalk at a level above the head of F. While crystal structures of other *Paramyxoviridae* family receptor-binding proteins have been solved in a tetrameric state [65,67,112] (in solution), in this study, direct visual evidence using subtomogram averaging shows no evidence of tetrameric forms of HN on the surface of authentic virions. (**Fig 2** and **S2 Fig**). Imposing different tetrameric references in our subtomogram averaging caused a divergence from the tetrameric reference back to the dimer densities. We show that there is a close association between HN and F, prior to receptor engagement, and, using subtomogram averaging, we did not observe HN in a heads-down conformation (**Fig 2**). While we cannot eliminate the possibility that, in some conditions, HN may exist in a heads-down conformation or as a tetramer, under the conditions used in this study (minimal perturbation and physiological temperatures), we did not observe a heads-down state on the surface of viral particles. It is theoretically possible that the enrichment of particles by binding to antibodies on the surface of cryo-EM grids could alter the organization of surface glycoproteins distant from the site of antibody interaction. To minimize these effects, cryo-EM grids were washed to remove excess antibody, so that only antibodies adsorbed to the carbon surface remain, prior to virus attachment, limiting virus-antibody binding to localized points of attachment. Furthermore, subtomogram averaging displayed uniform tight interaction between HN and F, indicating a stable unperturbed complex across the viral surface with distributed orientations of all sub-volume extracted particles (**S1B Fig**).

With the advances described here, it is now possible to image interactions between viral glycoproteins *in situ*, as the interacting proteins progress through their roles during entry. We can apply cryo-EM to dissect the stepwise process by which HN triggers F-mediated fusion when forming a complex with receptors on a target cell membrane. The target cell membranes used here–human erythrocyte fragments–were chosen because these membranes contain the sialic acid receptors used by HPIV3, as well as an authentic lipid composition, similar to typical plasma membranes. The preparation of human erythrocyte fragment membranes differs from the artificial liposomal preparation used for cryo-EM fusion studies of viruses, such as influenza [113–115]. F triggering occurs at 37°C but not at 4°C [34,116], which allowed us to use temperature to examine the receptor-engaged intermediate states of the HN-F complex, prior to initiation of fusion (**Fig 3**). Receptor engagement induced HN to trigger F at 37°C, activating fusion, and the F-interacting peptide then bound to the exposed heptad repeat region of F, blocking F’s refolding (**Fig 4**) and trapping the intermediates at an extended state [33]. By blocking F’s refolding after activation by HN, we can image HN and F interactions in the presence of different inhibitors that target distinct parts of the complex.

When viral particles were permitted to interact with target membranes at 4°C (a temperature at which receptor engagement can occur, but F does not undergo conformational transition), HN and F were observed with HN both free and receptor-engaged (**Fig 3**). Remarkably, HN appears less extended when receptor-engaged than when free. As shown in **Fig 3F**, the average distance between the viral membrane and the HN heads when HN is receptor-engaged is 138 Å, 25 Å shorter than when HN is not receptor-engaged, and this size difference does not seem to be due to steric constraints due to the target erythrocyte fragment membrane, as we can observe an approximately 27 Å gap between HN and the target membranes. One possible explanation for the decreased extension of HN could be image delocalization effects, due to the contrast transfer function (CTF) of the microscope. While all images were CTF-corrected during tomogram alignment and reconstruction, we cannot exclude that errors in CTF correction result in changes in density profiles. We do not observe visible halo effects in our images, so we are encouraged that our analysis is not likely adversely impacted by imaging distortions. A central question in this field has been how receptor engagement by HN leads to activation of an adjacent F. The measurements here suggest that receptor engagement leads to a shortening of HN, while the density of the globular domain of HN remains similar, and we propose that this shortening may affect the stalk of HN, a domain known to be important for activating F [8]. Future studies will address the question of whether the HN stalk may be bowing or compacting and/or altering its interaction with F during viral entry.

To characterize the intermediate state of F, we used a peptide corresponding to the C-terminal HR domain of F (VIKI-PEG_4_-chol) that prevents the intermediate F from collapsing into the post-fusion conformation. We observed elongated densities with diameters estimated to be between 19 and 30 Å that spanned from the viral membrane to the target erythrocyte fragment membrane (**Fig 4 and S4 Fig**). Recently, multiple intermediate states of influenza hemagglutinin were solved by cryo-EM, wherein the extended fusion trimer structure had an average diameter of 28 Å [87], similar to the average diameters of the elongated densities we observed here. These elongated densities can be seen clustered together (**Fig 4C–4E and S4 Fig**), possibly indicating a cascade of activation upon the triggering of one of more F molecules.

At 37 ^o^C, viral particles can fuse with target erythrocyte fragment membranes. We observed non-spherical viral particles that are likely to represent incorporation of the spherical particle into the target erythrocyte fragment membrane. The fused particles in this study generally have kinked membranes. While we have not seen such kinks in membranes at other viral-host interaction steps, we cannot entirely rule out that these kinks have occurred due to vitrification-related issues, such as sheer stress or osmotic effects, due to evaporation during sample blotting. We observed very few instances of the hemifusion intermediate state (**Fig 5F**); the incubation conditions (30 min at 37°C) might have contributed to failure to observe this state. Previously, influenza hemifusion states have been visualized by cryo-EM with the aid of hemifusion mutants or short incubation times, combined with lipid dyes used to identify these regions [45,117]. In future studies, applying those approaches to our system should allow us to capture hemifusion states of HPIV3 [45].

Our sub-tomogram averages show a stable interaction between HN and F in the prefusion state, as the two proteins would otherwise not be resolved together as a complex. Higher resolution of complexes on authentic viral surfaces should permit analysis of these interactions, especially in the HN stalk region, which has been shown to be integral for F activation, and will be necessary for understanding the relationship between HN and F prior to receptor engagement. Recombinant viruses that bear HNs with specific mutations that alter HN-F interaction or fusion promotion will be useful to examine the structural basis for functions of the fusion complex [8,32,42,118–120]. Sequentially imaging HN-F complexes with an altered primary sialic acid binding site on HN’s globular head, secondary binding site at HN’s dimer interface, or stalk, may uncover function of these domains. Experiments like those in **Figs 2–5** will reveal the mechanisms that underlie the biological impact of these residues.

## Materials and methods

### Virus growth and purification

Vero cells (African green monkey kidney cells) (ATCC, CCL-81) were grown in Dulbecco’s modified Eagle’s medium (DMEM) (Cellgro), supplemented with antibiotics and 10% fetal bovine serum, in a humidified 5% CO_2_ incubator. Cells were infected with a lab-adapted strain of HPIV3 [121] in Opti-MEM (Thermo Fisher) and incubated for 90 min [121]. Viral inocula were replaced with complete medium and returned to a humidified 5% CO_2_ incubator. Next, the cell culture supernatant fluid was collected and clarified by low speed centrifugation (1800 rcf for 10 minutes at 4°C). Clarified supernatant fluid was either used fresh or in some cases flash frozen and immediately stored at -80°C for later use. Clarified supernatant fluid for ultracentrifuged samples underwent further centrifugation (25,000 rpm for 240 min at 4°C in an SW28 rotor, Beckman L8-80M ultracentrifuge) through a 10 ml 30% (wt/vol) sucrose cushion in phosphate-buffered saline (pH 7.4). Titers for purified viruses were at least 1.00 x 10^7^ plaque forming units (PFU)/ml.

### Erythrocyte fragment membrane preparation

Human red blood cells (RBC) were used in experiments as a surrogate host target membrane. RBC were separated from the plasma with a low speed centrifugation (800 rcf for 5 min) and then washed at least 2 times with Dulbecco's phosphate-buffered saline (DPBS) (Gibco). The purified RBC were suspended in 10% DPBS medium and stored at 4°C for up to 5 days. For use in cryo-EM and cryo-ET experiments, red blood cells were extruded with 10 passes through a 400 nm filter and then subsequently through a 100 nm filter, using the Avanti lipid extruder. These surrogate host target membranes were used fresh within 24 hours of preparation.

### Chemicals and antibodies

Zanamivir (Acme Biosciences) was dissolved in Opti-MEM at a concentration of 50 mM and stored at -80°C. VIKI-PEG_4_-chol, the fusion inhibitory peptide, was produced, as previously described [33]. Briefly, peptides were produced by standard Fmoc-solid phase methods, and the cholesterol moiety was attached using displacement of an α-bromoamide. Bromoacetyl–PEG_4_-cholesterol was custom synthesized by Charnwood Molecular (UK). VIKI-PEG_4_-chol (5mM in DMSO) was kept at -20°C. Monoclonal anti-HPIV3 HN antibodies were custom elicited in rats (Aldeveron) using eGFP-HN cDNA, diluted in DPBS to 100 μg/ml and kept at 4°C.

### Cryo-electron tomography preparation

Lacey carbon gold grids, containing a continuous layer of thin carbon (Ted Pella), were plasma cleaned with Fischione M1070 Nanoclean on 70% power for 20 seconds with a 25% Oxygen, 75% Argon gas mixture. 8 μl drops, containing 100 μg/ml of the anti-HPIV3 HN antibody, were incubated on the grids for 10 minutes, then the grids were washed with DPBS to remove unabsorbed antibodies. For negative controls, we applied an antibody specific for measles H to the grids, and we did not observe viral particles on these grids. Next, grids were blotted and placed face-down in the Vero cell supernatant fluid, containing HPIV3 and/or host target erythrocyte fragment membrane preparations, in a 6-well plate. For samples containing zanamivir or fusion inhibitory peptides, these samples were incubated with the supernatant fluid or target erythrocyte fragment membranes, respectively. Plates were incubated for 30 min at 4°C with rocking. After incubation in the supernatant fluid, the grids were washed in cold DPBS 15 times. Grids were then plunge frozen in liquid ethane, using a Vitrobot (Mark IV; Thermo Fisher Scientific Co.).

### Cryo-electron tomography collection for subtomogram averaging

Vitrified grids were imaged using a Titan Krios 300 kV transmission electron microscope (Thermo Fisher Scientific Co.), equipped with a Gatan K2 direct detector and a Gatan Bioquantum energy filter set in zero-loss mode with a slit width of 15 eV. Movie images were captured at a 64,000x magnification for a 1.84 Å/pixel image size. Movie frames were acquired with Leginon software [122] with a 3.0 μm defocus and a ± 50 degree tilt and a total dose of 120 e^-^/Å^2^.

### Cryo-electron microscopy and tomography collection for HPIV3–erythrocyte membrane interactions

Vitrified grids were imaged using a Titan Halo 300 kV transmission electron microscope (Thermo Fisher Scientific Co.), equipped with a direct detection Gatan K3 camera with no energy filter. Images were captured at a magnification of 18000x and binned by a factor of 2, giving a pixel size of 3.46 Å at the specimen level. Images were acquired with SerialEM software [123] with a 3.5 μm defocus and either a single image with a total dose of 34 e–/Å^2^ or a tilt-series at 3° steps from 51° to -51° with a total dose of ~120 e^-^/Å^2^.

### Cryo-EM and cryo-ET image processing

All micrograph movies were aligned using WarpEM or MotionCorr2 [124]. Tomograms were reconstructed using Appion-Protomo [125]. All cryo-EM movie images were visualized using ImageJ, IMOD [126], and Chimera [127]. Distance measurements in **Fig 3** (n = 38), **Fig 4** (n = 3) and **S4 Fig** (n = 4) were obtained in ImageJ, using the plot profile function with a line width of 4 Å. Distances were exported into Excel, and averages were obtained. Surface density color representations were obtained from ImageJ’s 3D surface plot function, where the density is averaged from the neighboring pixels.

The subtomogram averaging process (outlined in **S4 Fig**) was performed using the Dynamo software package. Briefly, sub-volumes of the viral particle surfaces (160)^3^ Å were extracted from 2X binned tomograms. A round of reference free alignment was followed by centering all particles to an HN and F complex. A reference was then generated from the average of all sub-volumes and filtered to 30 Å. The data sets were subjected to two rounds of alignment in the presence of a mask, encompassing the HN and F complex. A third round of alignment included the membrane in the mask. Particles with the highest cross correlation were selected as the final density. Fourier Shell Correlation and Resmap [128] were both used to validate the final resolution. The number of particles included in the analysis, and the strategy utilized are summarized in **S1 Table.**

## Supporting information

S1 Fig Cryo-electron tomography statistics. (A) Schematic of pre-fusion sub-volume average workflow. (B) Orientation distribution of particles in the final sub-volume average. (C) Fourier shell correlations (FSC) of the final sub-volume average without (23.98 Å) and with (17.18 Å) a tight HN-F complex mask. (D) Resmap resolution of the HN-F complex measured with a tight HN-F complex mask.
(TIF)

S2 Fig Docking of tetramer structures in sub-volume average of prefusion HN-F complex. (A) Tetramer of PIV5 (PDB ID:1Z50) with both HN dimers in a heads-up conformation fitted into the final sub-volume average. One HN dimer in the HN tetramer completely lacks any density in the final sub-volume average. (B) Tetramer of PIV5 (PDB ID:4JF7) with one HN dimer in heads-up and the other dimer in a heads-down conformation fitted into the final sub-volume average.
(TIF)

S3 Fig Imaging interactions of HPIV3 and target erythrocyte fragment membranes. HPIV3 and target erythrocyte fragment membrane samples were incubated at 4°C prior to vitrification. (A-D) Contrast-inverted cryo-ET central Z-slices of HPIV3 interacting with target erythrocyte fragment membranes. Insets show enlarged regions of viral-target membrane interactions, where thin lines of density can be seen extending from the surface glycoproteins to the host membrane. (E-H) HPIV3 interactions with target erythrocyte fragment membranes in the presence of zanamivir to disrupt HN-receptor binding. Scale bars: (A-H) 50 nm.
(TIF)

S4 Fig Imaging interactions of HPIV3 and erythrocyte fragment membranes in the presence of a fusion inhibitory peptide. All HPIV3 and target erythrocyte fragment membrane samples were incubated at 37°C in the presence of a fusion inhibitory peptide (VIKI-PEG_4_-chol) prior to vitrification. (A-D) Contrast-inverted cryo-EM images of HPIV3 interaction with erythrocyte fragment membranes with insets below showing an enlarged region of viral-host interactions. Enlarged insets include representative lines where distance plot measurements were taken. Density line plots show widths at the half-maxima of densities. Scale bars: (A-D) 50 nm.
(TIF)

S5 Fig HPIV3 interactions with erythrocyte fragment membranes in the absence and presence of zanamivir. HPIV3 and target erythrocyte fragment membrane samples were incubated at 37°C in the presence of a fusion inhibitory peptide (VIKI-PEG_4_-chol) to lock F in an extended state, prior to vitrification. (A-C) Contrast-inverted cryo-EM images of HPIV3 interaction with target erythrocyte fragment membranes with VIKI-PEG_4_-chol and without zanamivir. Insets show enlarged regions of viral-host interactions. (D-F) HPIV3 interactions with erythrocyte fragment membranes with VIKI-PEG_4_-chol and with zanamivir to disrupt HN-receptor binding. Enlarged regions show target erythrocyte fragment membrane attachment remains where HN binding is blocked. Scale bars: (A-F) 50 nm.
(TIF)

S1 Table Cryo-ET data collection statistics. (TIF)

S1 Movie Tomogram of HPIV3 prior to receptor engagement. (see **Fig 2**)
(MP4)

S2 Movie Tomogram of HPIV3 and target erythrocyte fragment membrane incubated at 4°C. (see **Fig 3**)
(MP4)

S3 Movie Tomogram of the intermediate state of F captured with a lipid-conjugated peptide fusion inhibitory peptide. (see **Fig 4**).
(MP4)
