# Supplementary figures and images for: Human parainfluenza virus fusion complex glycoproteins imaged in action on authentic viral surfaces
Source: PLoS Pathog. 2020 Sep 21;16(9):e1008883. doi: 10.1371/journal.ppat.1008883 (PMC7529294; doi:10.1371/journal.ppat.1008883)

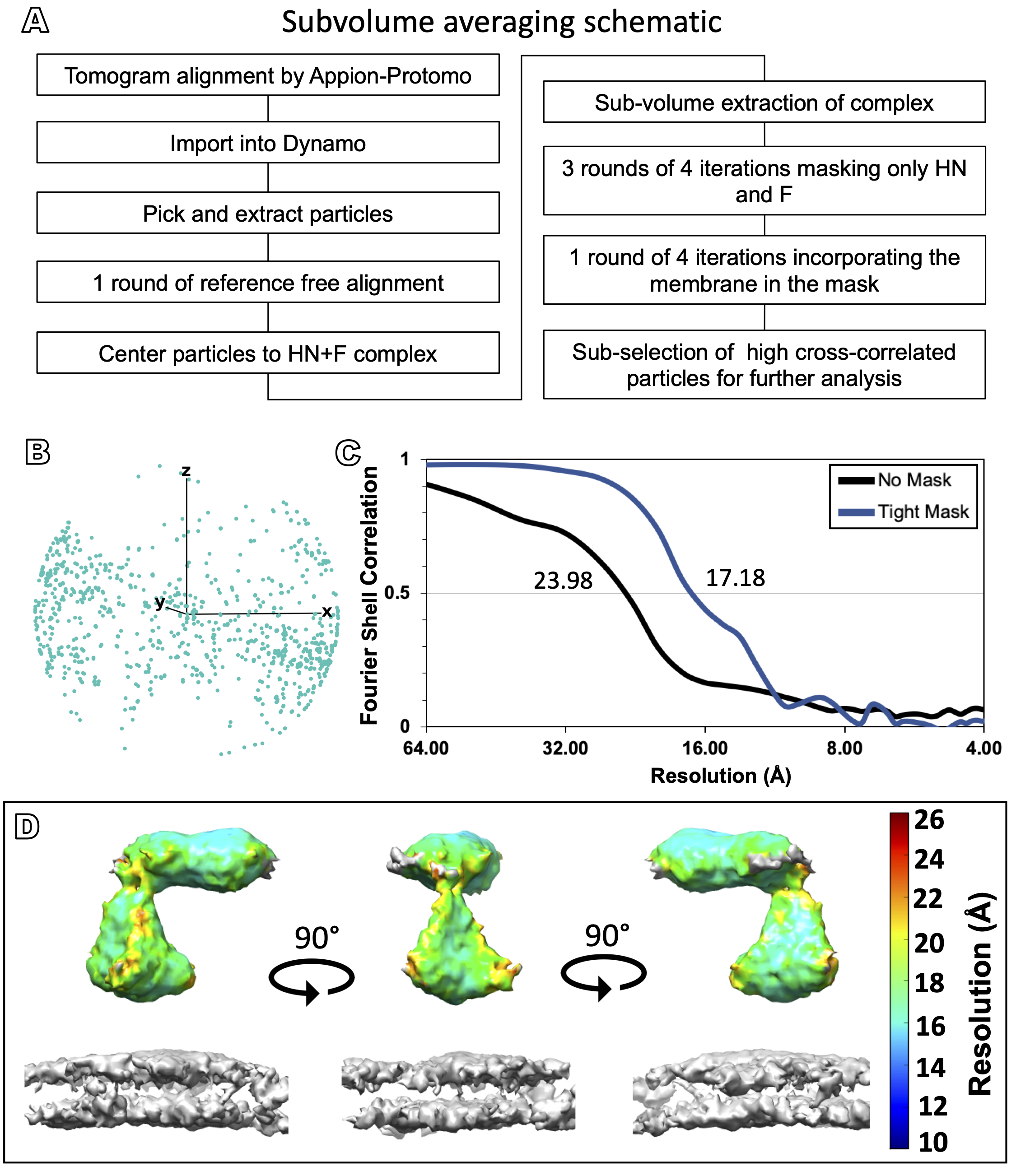

Supplement: S1 Fig — (A) Schematic of pre-fusion sub-volume average workflow. (B) Orientation distribution of particles in the final sub-volume average. (C) Fourier shell correlations (FSC) of the final sub-volume average without (23.98 Å) and with (17.18 Å) a tight HN-F complex mask. (D) Resmap resolution of the HN-F complex measured with a tight HN-F complex mask. (TIF) [file ppat.1008883.s001.tif]

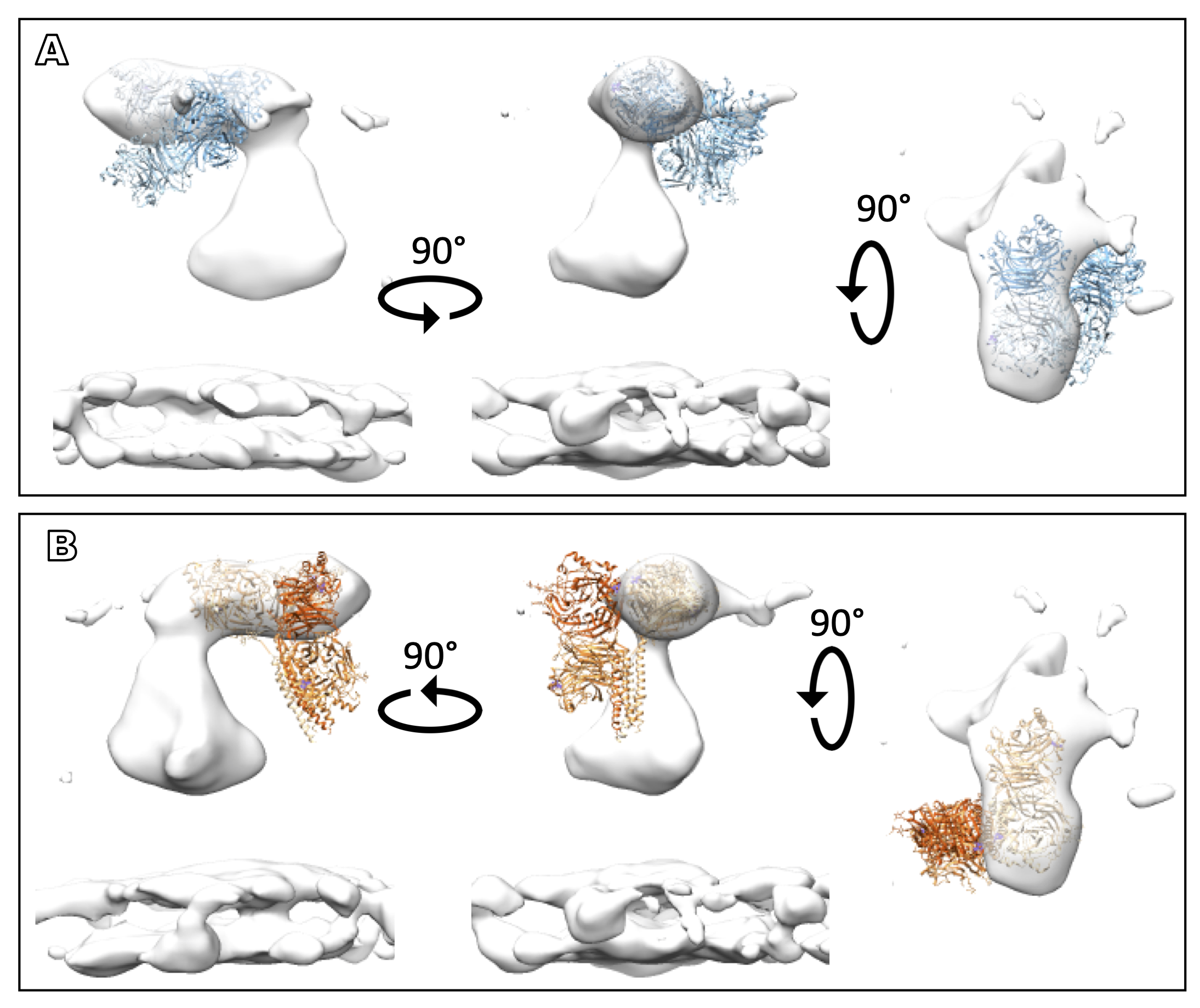

Supplement: S2 Fig — (A) Tetramer of PIV5 (PDB ID:1Z50) with both HN dimers in a heads-up conformation fitted into the final sub-volume average. One HN dimer in the HN tetramer completely lacks any density in the final sub-volume average. (B) Tetramer of PIV5 (PDB ID:4JF7) with one HN dimer in heads-up and the other dimer in a heads-down conformation fitted into the final sub-volume average. (TIF) [file ppat.1008883.s002.tif]

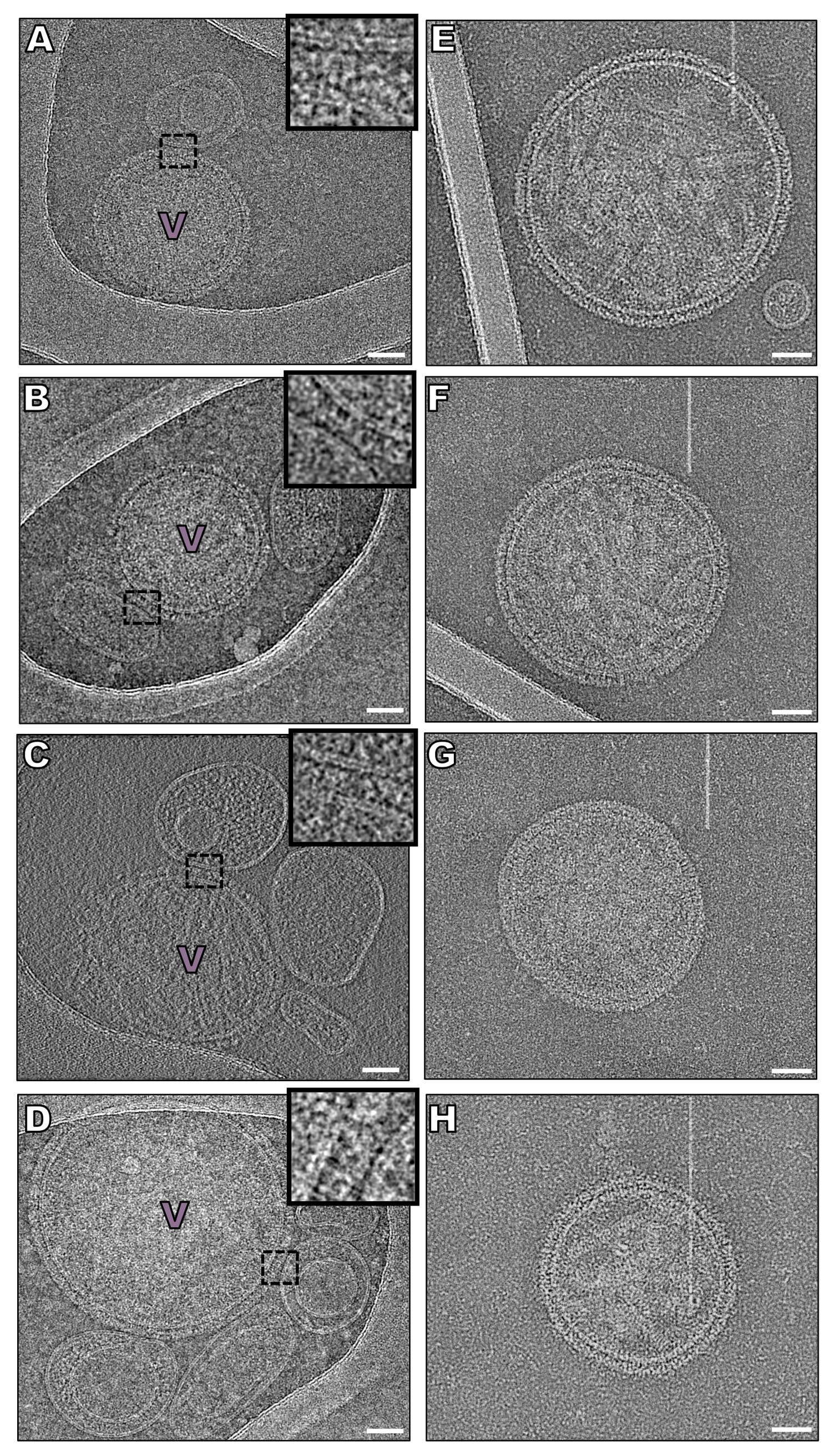

Supplement: S3 Fig — HPIV3 and target erythrocyte fragment membrane samples were incubated at 4°C prior to vitrification. (A-D) Contrast-inverted cryo-ET central Z-slices of HPIV3 interacting with target erythrocyte fragment membranes. Insets show enlarged regions of viral-target membrane interactions, where thin lines of density can be seen extending from the surface glycoproteins to the host membrane. (E-H) HPIV3 interactions with target erythrocyte fragment membranes in the presence of zanamivir to disrupt HN-receptor binding. Scale bars: (A-H) 50 nm. (TIF) [file ppat.1008883.s003.tif]

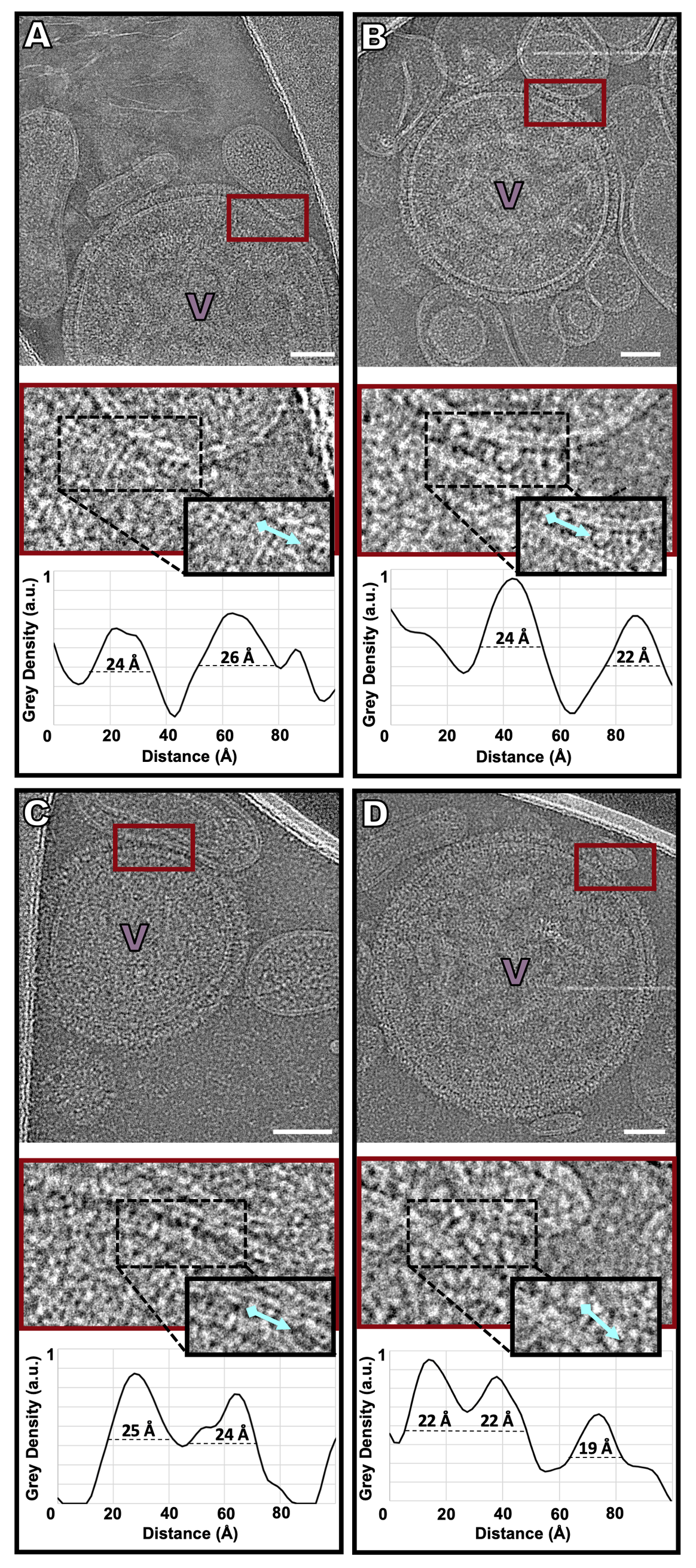

Supplement: S4 Fig — All HPIV3 and target erythrocyte fragment membrane samples were incubated at 37°C in the presence of a fusion inhibitory peptide (VIKI-PEG4-chol) prior to vitrification. (A-D) Contrast-inverted cryo-EM images of HPIV3 interaction with erythrocyte fragment membranes with insets below showing an enlarged region of viral-host interactions. Enlarged insets include representative lines where distance plot measurements were taken. Density line plots show widths at the half-maxima of densities. Scale bars: (A-D) 50 nm. (TIF) [file ppat.1008883.s004.tif]

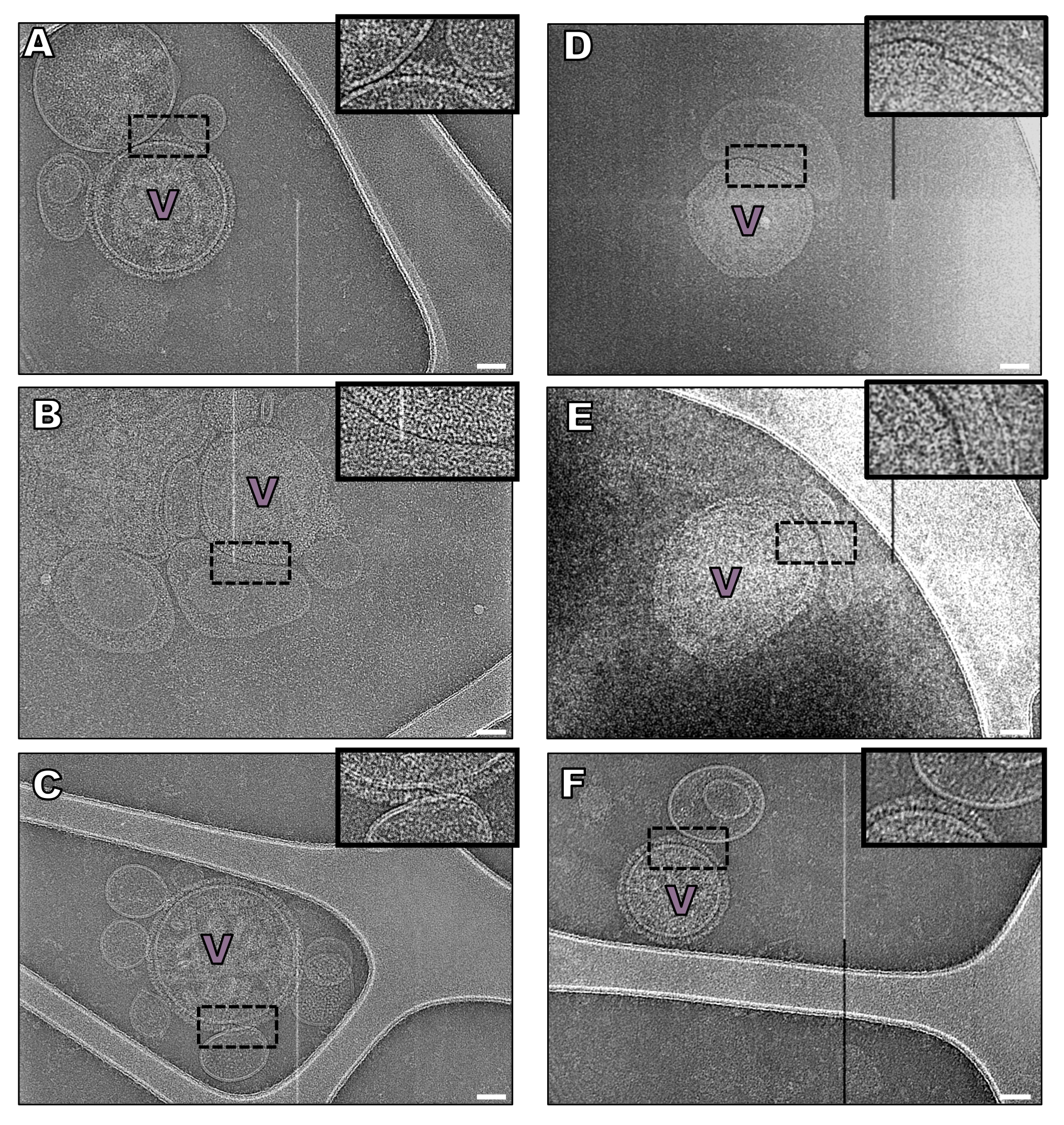

Supplement: S5 Fig — HPIV3 and target erythrocyte fragment membrane samples were incubated at 37°C in the presence of a fusion inhibitory peptide (VIKI-PEG4-chol) to lock F in an extended state, prior to vitrification. (A-C) Contrast-inverted cryo-EM images of HPIV3 interaction with target erythrocyte fragment membranes with VIKI-PEG4-chol and without zanamivir. Insets show enlarged regions of viral-host interactions. (D-F) HPIV3 interactions with erythrocyte fragment membranes with VIKI-PEG4-chol and with zanamivir to disrupt HN-receptor binding. Enlarged regions show target erythrocyte fragment membrane attachment remains where HN binding is blocked. Scale bars: (A-F) 50 nm. (TIF) [file ppat.1008883.s005.tif]

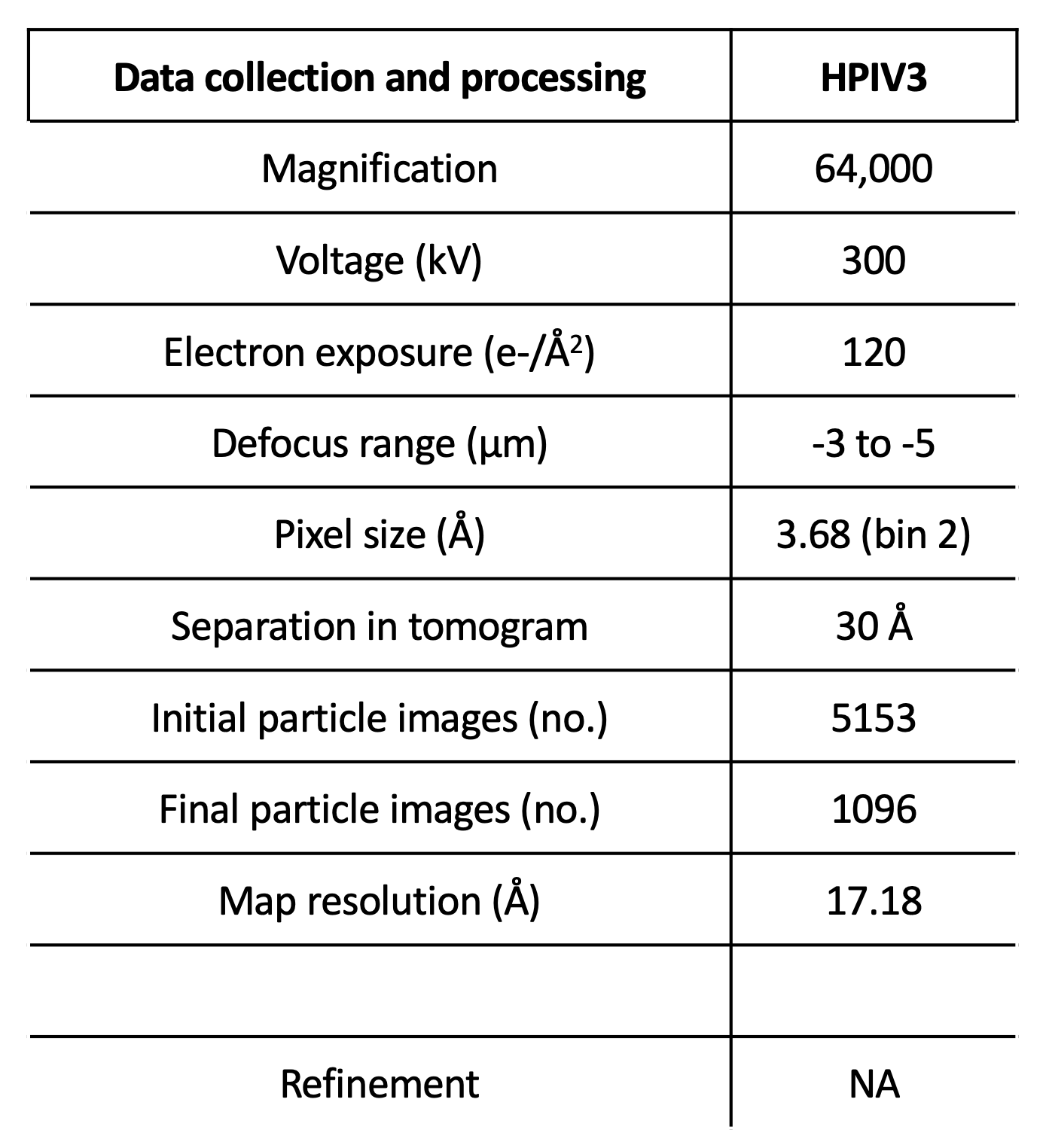

Supplement: S1 Table — (TIF) [file ppat.1008883.s006.tif]
